# Cosmeceuticals in acne vulgaris: from mechanism of action to clinical application

**DOI:** 10.1093/skinhd/vzaf104

**Published:** 2026-01-09

**Authors:** Tamara N Searle, Firas Al-Niaimi, Faisal R Ali

**Affiliations:** Department of Dermatology, Imperial College London Healthcare NHS Trust, London, UK; Department of Dermatology, Aalborg University Hospital, Aalborg, Denmark; School of Medicine and Dentistry, University of Lancashire Medical School, Preston, UK

## Abstract

The use of cosmeceuticals in acne vulgaris is becoming increasingly prevalent with many over-the-counter formulations becoming part of patients’ routine skincare. Cosmeceuticals are often used successfully as an adjunctive therapy to reduce the side effects of traditional prescriptions and improve compliance. This is a review to support the use of cosmeceuticals in acne, including retinol, retinaldehyde, benzoyl peroxide (BPO), azelaic acid, beta hydroxy acids, alpha hydroxy acids, niacinamide, zinc, tea tree oil and green tea. There is most evidence, in human clinical trials, to support the use of topical retinol, BPO and azelaic acid. Further research with large-scale robust human clinical trials are required to go beyond *in vitro* studies. Most research has focused on mild-to-moderate acne and few studies have looked at the use of cosmeceuticals in more severe acne. Overall, adjunctive treatment with cosmeceuticals might reduce the side effect profile of standard therapies such as dryness, itching, scaling and erythema, promoting treatment compliance and improving acne outcomes.

## Teaser

The use of cosmeceuticals in acne vulgaris is becoming increasingly prevalent with many over-the-counter formulations becoming part of patients’ routine skincare. Cosmeceuticals are often used successfully as an adjunctive therapy to reduce the side-effects of traditional prescriptions and improve compliance. This is a review to support the use of cosmeceuticals in acne including: retinol, retinaldehyde, benzoyl peroxide, azelaic acid, beta hydroxy acids, alpha hydroxy acids, niacinamide, zinc, tea tree oil and green tea.

Cosmeceuticals are topical treatments with biologically active properties that may provide therapeutic effects without the requirement for a doctor’s prescription. Cosmeceuticals can be used as an adjunctive treatment to reduce the side effect profile of established treatments such as retinoids or antibiotics, improving patient compliance. Topical retinoids are derivatives of vitamin A and are effective in treating both comedonal and inflammatory acne by binding to retinoic acid receptors (RARs), reducing keratinocyte proliferation and exhibiting anti-inflammatory effects. Benzoyl peroxide (BPO) is an oxidative agent that is bactericidal against *Cutibacterium acnes*, and possesses anti-inflammatory and anti comedonal properties by interfering with sebaceous secretion and disrupting cellular processes. National Institute for Health and Care Excellence (NICE) guidelines recommend azelaic acid in combination with oral antibiotics for moderate-to-severe acne or as a second-line monotherapy option if adapalene/BPO combinations are not tolerated. Alpha and beta hydroxy acids (AHAs and BHAs) exfoliate the skin by removing dead skin cells, with AHAs primarily improving surface texture and pigmentation, while BHAs like salicylic acid (SA) penetrate pores to target oily skin and reduce inflammation. Niacinamide, a water-soluble form of vitamin B3, reduces acne symptoms by decreasing sebum production, reducing inflammation and strengthening the skin barrier through its antibacterial and anti-inflammatory properties. Zinc is a micronutrient with anti-inflammatory, antimicrobial and anti androgenic properties and is thought to work in acne by regulating the immune response, inhibiting *C. acnes* growth and reducing the production of inflammatory cytokines. Tea tree oil, derived from *Melaleuca arternifolia*, has antimicrobial and anti-inflammatory properties, making it effective against *C. acnes* and augmenting the efficacy of treatments like azelaic acid in acne management. Green tea, rich in polyphenols like epigallocatechin gallate (EGCG), possesses antioxidant, anti-inflammatory and antimicrobial properties, making it beneficial in reducing acne symptoms and improving overall skin health. Stronger clinical support exists for the use of retinol, BPO and azelaic acid, while other cosmeceuticals such as niacinamide, zinc, tea tree oil and green tea could be promising adjuncts, although there is less robust evidence to support their use. Many current studies use formulations with multiple ingredients, making the assessment of individual component efficacy challenging; larger randomized controlled trials (RCTs) with standardized outcome measures are required, and most research is based on mild-to-moderate acne, demonstrating a gap in the clinical evidence for studies on the use of cosmeceuticals in more severe acne. The portmanteau ‘cosmeceuticals’, an amalgam of cosmetics and pharmaceuticals, are ‘topical preparations… with performance characteristics suggesting pharmaceutical action’.^1^ These items are typically available without prescription and not subjected to the rigorous scrutiny of the Medicines and Healthcare products Regulatory Agency. Furthermore, cosmeceuticals are often combined with other agents, posing a greater challenge in discerning which active ingredient is contributing to the therapeutic benefit. Presently, the field of cosmeceuticals is rapidly expanding with the use of personalized skin care in acne vulgaris.^2^

In 2023, the UK beauty and personal care sector contributed £27 billion to the economy, £13.5 billion directly, emphasizing the growing commercial and clinical relevance of cosmeceuticals in dermatology, particularly in acne vulgaris.^3^ Although, most research exploring the use of cosmeceuticals has been *in vitro*, there is ongoing human trial data emerging supporting their use in the treatment of various skin ailments, including acne. In this review, we investigate cosmeceuticals in acne, including retinol, BPO, azelaic acid, BHAs, AHAs, niacinamide, zinc, tea tree oil and green tea. We explore the current evidence with a focus on their mechanisms of action, clinical efficacy and implications for practice.

## Methods

Using the key words ‘acne vulgaris’, ‘alpha hydroxy acid’, ‘azelaic acid’, ‘benzoyl peroxide’, ‘beta hydroxy acid’, ‘green tea’, ‘niacinamide’, ‘retinal’, ‘retinaldehyde’, ‘retinol’, ‘tea tree oil’ and ‘zinc’, we searched PubMed for relevant literature. Our review was conducted in April 2025. The search encompassed the period spanning database inception until April 2025. The level of evidence was evaluated and selected according to the highest level, working our way downwards. Using the Oxford Centre for Evidence-Based Medicine 2011 guidance, we analysed the evidence based on its strength (from level 1 to level 5), with systematic reviews and meta-analyses considered first and RCTs second, cascading down to a weaker level of evidence such as case reports. Included studies are summarized in Table 1.

**Table 1:** Summary table of included studies

Agent	Sample size (*n*; pooled)	Intervention	Duration	Outcomes	Side effects	Limitations	Evidence level	Reference(s)
Retinol (monotherapy/combination)	244	Retinsphere post isotretinoin; various combinations with various combinations of hydroxypinacolone retinoate, retinol (0.5–1%), papain, azelaic acid/SA/GA/mandelic acid/niacinamide/BPO (2.5–4%)/niacinamide/7-dehydrocholesterol, niacinamide/BPO	12 weeks–12 months	Reduced lesion counts, improved IGA, QoL, some showed lower irritation vs. BPO, 15% relapse rate with retinsphere after isotretinoin	Dryness, erythema, peeling depending on subtype, formulation and concentration	Small/open-label trials, short durations, heterogeneity	High	^9–15^
Retinaldehyde (monotherapy/combination)	2412	Retinaldehyde 0.1% in nanoemulsion or various combinations of retinaldehyde 0.1% + GA 6%/efectiose 1%/topical erythromycin 4%	1–3 months	Reduced comedones, papules, pustules, microcysts, scarring, pigmentation	Dryness, erythema, peeling depending on subtype, formulation and concentration	Mostly observational studies +nonrandomized, short duration and small sample sizes	High	^16–22^
BPO	30 831	BPO 2.5–20% vs. vehicle	8–12 weeks	Significant IGA and lesion reduction	Pruritus, skin burning and bleaching of clothes	Heterogeneity across studies in Cochrane review, lack of long-term data on sustainability, potential safety risk	High	^25,27–29^
Azelaic acid	>750	Azelaic acid 15–20% cream/gel, alone or compared with placebo, oral contraceptive pill, BPO/clindamycin, tretinoin, adapalene, spironolactone	3–9 months	Reduced lesion counts, reduced inflammatory lesions, improved global acne scores. No significant difference between azelaic acid and tretinoin or BPO	Erythema, dryness, peeling, pruritus, and burning	High heterogeneity in formulations, durations and comparators, mostly open-label designs, tolerability issues with twice-daily use	Moderate	^37–49^
AHAs/BHAs	1860	GA, SA, mandelic acid used with/without lasers	2–10 weeks	SA + laser improved IGA and comedones vs. chemical peel; no major difference among AHA types	Skin dryness, redness or irritation and photosensitivity	Small, split-face trials, mostly cosmetic endpoints	Moderate	^57–60^
Niacinamide (monotherapy/combination)	446	Niacinamide 4–5% gel vs. clindamycin 1–2% gel. Adapalene/BPO + niacinamide 2–5% in dermocosmetic regimens with adapalene/BPO or isotretinoin or erythromycin; also in complex formulations with retinol, SA and GA	8 weeks–6 months	Comparable to clindamycin with fewer concerns of microbial resistance; improved tolerability and adherence; reduced xerosis	Mild itching, burning, mild dermatitis and greasy skin	Small trials, short follow-up, variable formulations	Moderate	^11,82–89^
Zinc (monotherapy/combination)	2798	Oral zinc, oral zinc + low-dose isotretinoin, topical zinc + topical erythromycin, nicotinamide, SA cleansers	3–12 weeks	Significantly lower baseline mean (SD) zinc levels in patients with acne [96.308 (4.053) μg dL^–1^] than in control participants [102.442 (3.744) μg dL^–1^] (*P* = 0.041); zinc improved inflammatory papule count; combinations showed modest improvements in lesion count (especially noninflammatory); no added efficacy with isotretinoin but fewer side effects, significant reductions in noninflammatory lesion counts in those treated with formulation of zinc, adapalene and niacinamide compared with adapalene alone; no statistically significant difference between combinations of erythromycin and zinc compared with erythromycin alone	Some reports of gastrointestinal symptoms such as nausea, vomiting or abdominal pain but no statistically significant difference found compared with comparator	Small, short-term studies limits statistical power nonrandomized/open-label design in some studies, heterogeneity in dosing and formulations makes optimal dosing unclear	Moderate	^96–100^
Tea tree oil	284	Tea tree 5% oil vs. BPO 5%; tea tree oil + adapalene; tea tree oil + propolis + aloe vera vs. erythromycin	12 weeks	Comparable efficacy to BPO with fewer side effects; significant reduction in total, inflammatory and noninflammatory lesion counts when combined with adapalene; tea tree oil + propolis + aloe vera improved erythema scars + acne severity index compared with topical erythromycin; tea tree oil face wash reduced lesion counts and reduced mean IGA score	Peeling, dryness and scaling	Small sample sizes, delayed onset of action vs. BPO, heterogeneous formulations, confounding from combination therapies	Low	^101,104–107^
Green tea extract	550	Topical 2–5% green tea extract, tea polyphenols, polyphenol-based hydrogels, blends of green tea + lotus), sonophoresis + green tea, bamboo extract and lactic acid	4–12 weeks	Topical green tea significantly reduced inflammatory (−9.38, 95% CI −14.13 to −4.63) and noninflammatory lesions. Comparable efficacy to clindamycin 1%. Some evidence of sebum reduction in acne vulgaris	Mild gastrointestinal effects reported relating to oral green tea extract, and temporary irritation and itching was associated with topical green tea extract application	Small heterogeneous trials, inconsistent formulations, combination therapies, limited long-term data, most trials lacked acne recurrence data and standardized endpoints	Low	^114–122^

AHA, alpha hydroxy acid; BHA, beta hydroxy acid; BPO, benzoyl peroxide; CI, confidence interval; GA, glycolic acid; IGA, Investigator Global Assessment; QoL, quality of life; SA, salicylic acid.

## Topical retinoids

Topical retinoids are derivatives of vitamin A and have been the mainstay of acne treatment since tretinoin was first approved in 1971 by the Food and Drug Administration.^4^

Topical retinoids bind to RARs in the nucleus, thereby altering downstream gene transcription. There are three RAR subtypes, with different retinoid formulations targeting different RAR subtypes.^4^ Ultimately, retinoids act to reduce the proliferation of keratinocytes, stimulating keratinocyte differentiation to normalize the desquamation process. Topical retinoids exert anti-inflammatory effects by inhibiting Toll-like receptors (TLRs), leucocyte migration and the activator protein-1 (AP-1) pathway, thereby reducing nitric oxide production and cytokine release, and suppressing cell inflammation overall.^5^ They have benefits for comedonal and inflammatory acne.^6^ Retinoids are synthetic and natural analogues of vitamin A. The alcohol end group of retinols can be oxidized to form retinaldehyde (retinal), which can be subsequently oxidized to retinoic acid. Given the varying potencies, retinol and retinaldehyde (at lower concentrations) are available ‘over the counter’ in the UK, but retinoic acid requires a prescription.^7^ As our review is of cosmeceuticals, we have focused on the efficacy of retinol and retinal on acne, which are both available directly to consumers.

### Retinol monotherapy

In a small study, retinol and oligopeptide-loaded lipid nanocarriers were found to significantly reduce sebum secretion levels.^8^ The efficacy and safety of a retinsphere formulation – containing retinol encapsulated in glycospheres (Biretix Gel^®^; Cantabria Labs, Madrid, Spain) – were evaluated as a maintenance treatment after oral isotretinoin use in 40 patients. The results showed a relapse of acne lesions in only 15% of participants, with good tolerability.^9^

### Retinol combination

Ninety-eight patients with mild-to-moderate acne were treated with a combination of hydroxypinacolone retinoate 0.1% (synthetic esther of 9-*cis*-retinoic acid), retinol 1% in glycospheres and papain 2% in glycospheres, and had a 41% mean reduction in their Global Acne Grading System count after 12 weeks of use,^10^ with similar formulations containing retinol encapsulated with glycolic acid (GA), SA and niacinamide showing promising results in patients with mild acne.^11^ Smaller, lower-quality studies have explored the use of retinol in acne. A prospective split-face RCT evaluated a three-step routine of azelaic acid, SA and graduated retinol vs. BPO over a 12-week period in patients with ­mild-to-moderate acne, finding a similar reduction in total acne lesion count numbers and preferred user tolerability in the three-step group (*n* = 37).^12^ BPO 2.5% and topical retinol was applied to 33 patients with mild-to-moderate acne, with a significant reduction in global total acne found from baseline to week 12, with significant improvements in Investigator’s Global Assessment (IGA) of acne severity scores and quality of life scores without any increases in skin irritation.^13^ Combinations of BPO 4%, retinol 0.5%, mandelic acid 1% and lactobionic acid 1% also have been found to be effective and well tolerated in mild acne.^14^ Combinations of topical niacinamide, retinol and 7-dehydrocholesterol were found to downregulate various pro inflammatory molecules, which correlated with clinical response.^15^

### Retinaldehyde monotherapy

A study involving 23 patients investigated the effects of a topical retinaldehyde-loaded niosome nanoemulsion on mild-to-moderate acne, revealing a significant reduction in both closed and open comedone counts after 2 weeks.^16^

### Retinaldehyde combination

Retinaldehyde 0.1% combined with GA 6% was well tolerated and found to significantly decrease inflammatory acne lesions in two separate studies of over 2000 patients,^17,18^ while a similar combination significantly reduced global scarring score and hyperpigmentation.^19,20^ This was also observed when retinaldehyde 0.1% and GA 6% were combined with efectiose 1%.^21^ Topical erythromycin 4% and retinaldehyde 0.1% combinations significantly reduced papules, pustules, comedones and microcysts.^22^

### Side effect profile

The side effects of retinol and retinaldehyde include dryness, erythema and peeling, depending on the subtype, formulation and concentration. Side effects appear to increase in severity with increasing concentration.^23^ Retinoid concentration, vehicle formulation, type of retinoid used and skin sensitivity are all thought to influence tolerability.^24^ Overall, topical retinoids are an established treatment in the treatment of acne, with a high level of evidence to support their use. Different formulations and combination therapies allow for flexibility in tailoring treatments to patient’s individual skin type in terms of oiliness and dryness, leading to increased adherence. Over-the-counter cosmeceuticals could be useful for maintenance treatment after prescribed courses of topical retinoids and systemic isotretinoin, with fewer side effects or monitoring requirements. Patient education is essential in retinoid use, with gradual titration of dosage application depending on skin reaction. We acknowledge that the currently available studies have small sample sizes and are often open label, emphasizing the requirement for robust large-scale RCTs. It is also important to note that most studies investigating retinol or retinaldehyde are limited to mild-to-moderate acne, and their role in moderate-to-severe acne requires further exploration.

## Benzoyl peroxide

BPO is an oxidative agent that exhibits bactericidal properties against *C. acnes*, along with anti-inflammatory and comedolytic effects. Moreover, being lipophilic, it generates benzoic acid and reactive oxygen species, which interfere with sebaceous gland secretion, and disrupt protein and nucleotide synthesis, as well as mitochondrial function.^24^

A Cochrane review of 60 RCTs found that BPO significantly outperformed placebo in reducing inflammatory and noninflammatory lesion counts. The review noted considerable heterogeneity and variability in outcome measures across studies, making pooled quantitative estimates difficult. No or little significant difference was observed between BPO and adapalene or clindamycin, underscoring BPO’s role as a first-line, cost-effective alternative to prescription treatments.^25^ Various concentrations of BPO exist, ranging from 2.5% to 20%, with gel being the most common formulation. BPO has been used in combinations with adapalene, clindamycin and erythromycin, but these combinations are available via prescription only. BPO’s use with topical and oral antibiotics is thought to aid in preventing antibiotic resistance.^26^ In two RCTs of over 400 patients, when compared with vehicle treatment BPO demonstrated significant improvements in patients’ IGA success scores,^27,28^ with reductions in inflammatory and noninflammatory lesions after 12 weeks of treatment.^26,28^

While different concentrations of BPO have been investigated, including 2.5%, 5% and 10%, the 2.5% formulation has been shown to be more efficacious than the higher concentrations in reducing inflammatory lesion count and with fewer side effects compared with the 10% formulation.^29^

### Side effects

In terms of side effects, BPO can cause erythema, pruritus and skin burning and bleaching of clothes, which might lead to treatment discontinuation. A recent report raised concerns regarding the possibility of BPO products thermally decomposing to benzene when exposed to raised temperatures, potentially leading to malignancy.^30^ This has been disputed by other researchers, but, despite this, several BPO-containing products have been recalled in the USA.^31^ The Cochrane review supports BPO’s efficacy, but significant heterogeneity across included studies, and, as a result, the lack of a pooled effect size limits the generalizability of its findings, emphasizing the need for robust clinical trials with standardized methodologies. The noninferiority observed between BPO and prescribed agents may over simplify clinical decision-making, where combination regimes or patient factors are likely to have a role in the treatment decision. Long-term data on the sustainability of BPO’s treatment results and relapse rates are lacking, and the emergence of potential safety risks warrants further exploration of postmarketing surveillance data.

## Azelaic acid

### In vitro

Azelaic acid has antimicrobial and anti-inflammatory properties that inhibit mitochondrial metabolism.^32,33^ Azelaic acid modifies follicular epidermal hyperproliferation, and concentrations of 15% have superseded the 20% formulations that caused dry skin and irritation. NICE guidelines recommend azelaic acid in combination with oral antibiotics for ­moderate-to-severe acne or as a second-line monotherapy option if adapalene/BPO combinations are not tolerated.^34^ While the European evidence-based guidelines from 2016 recommend azelaic acid for mild-to-moderate papulopustular acne alone or in combination,^35^ the US guidelines recommend exploiting azelaic acid’s benefits in postinflammatory hyperpigmentation in people with Fitzpatrick skin phototypes IV–VI.^36^

### Human studies

A systematic review and meta-analysis found evidence to support the superiority of azelaic acid over a vehicle in reducing lesion counts and improving global acne scores. However, no significant difference was observed between azelaic acid and tretinoin, or between azelaic acid and BPO. Additionally, the comparison between azelaic acid and adapalene showed inconsistent results.^37^ No pooled quantitative outcomes were reported likely due to significant heterogeneity in study designs, outcome measures and formulations. Our review identified five RCTs and three open-label clinical trials that examined the use of azelaic acid in treating acne vulgaris. In the largest RCT (*n* = 220), BPO 3% combined with clindamycin 1% once daily was found, after 12 weeks, to be more effective than 20% azelaic acid cream applied twice daily.^38^ The study showed a significant difference between the two treatments in terms of reduction in inflammatory lesions, with BPO and clindamycin showing a median decrease of 52.6%, compared with a 38.8% reduction for azelaic acid (*P* = 0.0004). Additionally, total and inflammatory lesion counts, as well as tolerability, were notably different, with the azelaic acid group experiencing reduced tolerability (*P* < 0.0001). The azelaic acid group also reported significantly more side effects, including pruritus, pain, erythema and dryness, likely due to the twice-daily application.^38^ In two RCTs, azelaic acid proved more effective than a placebo, especially in addressing the inflammatory components of acne (*n* = 116).^39,40^ In another trial, 55 patients with acne treated with azelaic acid 15% twice daily for 9 months showed significantly better results in controlling both inflammatory and total lesions compared with those who used azelaic acid 15% for 3 months followed by 6 months of daily adapalene 0.1% gel.^41^ In another study (*n* = 38), the use of azelaic acid 15% gel in combination with oral contraceptives (drospirenone and ethinylestradiol) led to a significant decrease in TLR-2 levels in women with adult acne.^42^ This combination may exert anti-inflammatory effects by influencing TLR-2, as determined through immunohistochemistry and histomorphometric analysis.^43^ Three open-label clinical trials demonstrated that azelaic acid was effective in treating acne vulgaris, with improvements seen in clinical assessments, IGA scores and Dermatology Life Quality Index (DLQI; *n* = 291).^44–46^ Meanwhile, azelaic acid 20% chemical peel demonstrated long-term sebostatic action in 27 patients with acne,^47^ and azelaic acid was found to be comparable to tranexamic acid for acne-related postinflammatory hyperpigmentation.^48^ Further, a prospective RCT of adult women with acne found azelaic acid 15% to be comparable to spironolactone in terms of acne quality of life scores.^49^ Overall, the side effects associated with azelaic acid included erythema, dryness, peeling, pruritus and burning, although they were mild and mostly resolved by the end of the study period. The evidence supports azelaic acid’s superiority over placebo, but azelaic acid appears less effective than BPO and clindamycin. Significant heterogeneity in formulation, outcome measures and treatment duration, as well as methodological limitations such as open-label designs and small sample sizes across studies, limit the generalizability of the included studies’ findings. Robust standardized head-to-head trials are needed to clarify the role of azelaic acid compared with other prescribed agents.

### Hydroxy acids

Hydroxy acids (HAs) are naturally derived organic acids and include AHAs (GA from sugar cane; lactic acid from milk and fruits), BHAs (citric acid from citrus fruits; SA from willow bark) and poly HAs.^50^ HAs have keratolytic properties and antioxidant properties; they also increase epidermal turnover, making them useful in the dermatologist’s armamentarium for treating acne.^51^ In lower concentrations, HAs supplement over-the-counter skin creams, but in higher concentrations they can be used in chemical peels for the treatment of acne. Chemical peels work by inducing skin resurfacing, generating a new epidermal layer of the dermal tissue.^52^

### Beta hydroxy acids

BHAs are thought to aid exfoliation, are comedolytic and reduce inflammation. They are lipid soluble, with the most reported example being SA.^53^ At lower concentrations (1–6%), daily SA topical creams and gels have been used in acne; at higher concentrations (30%), SA peels used sporadically have been effective, with a good side effect profile in mild-to-moderate acne.^54^ SA can improve acne, scars, skin tone and texture, and can eliminate dead skin cells and reduce oil production; it works best at a pH of 3–4. Side effects include skin dryness, redness or irritation and photosensitivity.^55^ When combined with other treatments such as BPO it can also be more effective in treating acne.^56^ A combination of SA peel with pulsed-dye laser was shown to significantly improve IGA scores compared with chemical peel alone treatment (*P* = 0.003) in a split-face RCT (*n* = 19).^57^ In a separate study, a combination of 30% supramolecular SA and CO_2_ laser was superior to laser alone in reducing comedone distribution (*P* < 0.001).^58^ Trials comparing GA 35% peels with SA 20%–mandelic acid 10% peels showed no statistically significant differences between the treatments and there was no clear difference when SA was compared with topical tretinoin therapies.^59^ HAs are found in increasing numbers and combinations in over-the-counter, online and prescription formulations. Further research is required to establish the most efficacious combinations. Currently available data are from studies with small sample sizes over short time periods and are often conducted *in vitro*.^60^

### Alpha hydroxy acids

AHAs work on the skin’s surface to promote cell turnover,^53^ and the effectiveness of AHAs is dependent on pH, concentration and exposure time.^61,62^

### Monotherapy

As discussed, various concentrations of GA can be used. At concentrations of 5–15%, GA can be used in home skincare products to promote stratum corneum exfoliation, skin hydration and dermis regeneration. At concentrations of 20–35%, GA can be used in superficial peels for patients with oily skin types, while at concentrations of 50–70% it can be used in deeper peels by clinicians, inducing inflammation and stimulating dermis regeneration by producing collagen and elastin,^63^ assisting with the treatment of scars. At low concentrations, GA has shown statistically significant improvements in facial porphyrins and slight improvements in residual spots and erythema.^64^ A 2013 review^65^ discussed the use of GA in acne, finding that there was a significant reduction comedones, papules and pustules, as well as an improvement in skin texture, with consistent use of 35% and 50% GA peels once every 3 weeks for 10 weeks. GA 70% has been found to be better tolerated than Jessner’s solution (resorcinol 14%, lactic acid 14% and SA 14%, in an alcohol base) in people with Fitzpatrick skin type IV.^66^ Smaller, lower-quality studies have explored the use of GA in acne. However, SA peels were better tolerated when compared with GA peels in a split-face study after 20 patients were treated with peels biweekly for 6 treatments.^67^ In a separate study of 41 patients with Fitzpatrick skin types III–V, there was a significant reduction in the number of comedones and papules/pustules in patients with mild-to-moderate acne after GA peel, but there was no response in patients with nodulocystic acne.^68^ At high concentrations in peeling agents, a disruption of the corneocytes of the skin barrier was identified, leading to irritation in animal models, whereas at lower concentrations GA demonstrated anti-inflammatory effects through epigenetic mechanisms.^62^ In a further study, a ­dose-dependent response was demonstrated in collagen production in human skin explants topically treated with GA formulations of 8%, 10%, 15% or 25%.^69^ In a separate study comparing GA concentrations, GA 20% was found to be more effective at managing patients with acne with large pores, whereas GA 5% was more effective at managing inflammatory lesions. This could allow tailoring of concentrations of GA formulations depending on acne type and severity.^70^ The degree of antibacterial activity of GA on *C. acnes* was found to be most potent at pH 3, which is thought to work by disrupting bacterial cell membranes. The authors argue that much lower concentrations GA (as low as 0.2%) could be employed at this pH in over-the-counter preparations.^71^ Mandelic acid is another AHA shown to have efficacy in improving inflammatory lesions in mild-to-moderate acne with fewer side effects when compared with SA.^72^

### Combination therapy

GAs have often been combined with other active ingredients, including azelaic acid, tretinoin, clindamycin^73^ and adapalene, to produce a synergistic, more efficacious therapy.^74^ In a prospective study of 66 patients with acne-­related disorders, a serum of SA and GA was used by patients over a 2-week period. Over 90% of participants self-reported significant overall improvements in comedonal and cystic acne, with 70–80% reporting decreases in oiliness and skin texture, which was consistent with physical examination findings.^75^ A meta-analysis of 18 RCTs (*n* = 1435) found that AHAs were most efficacious when combined with intense pulsed light (IPL) compared with control [odds ratio (OR) 4.24, 95% confidence interval (CI) 2.66–6.74; *P* < 0.01]. AHA combined with IPL was more efficacious than AHA alone (OR 4.10, 95% CI 2.12–7.91; *P* < 0.01) and IPL alone (OR 4.02, 95% CI 2.25–7.16; *P* < 0.01) with no significant difference in adverse reactions between the combined and control group.^64^ Overall, the current evidence base for HAs is limited by methodological inadequacies. Although studies consistently report positive outcomes for SA and GA, particularly in combination treatment, most trials have limited generalizability due to their small sample sizes, short durations and open-label designs, with a lack of standardization in formulation making comparisons across studies challenging. Rigorous, large-scale RCTs are required to clarify their isolated efficacy and ideal treatment combinations.

## Niacinamide

Niacinamide (nicotinamide) is a water-soluble isotope of vitamin B3 (niacin). Niacinamide is thought to improve symptoms of acne vulgaris with its anti-inflammatory and anti bacterial profile, and its ability to reduce sebum production.^76^ Niacinamide has a role in neutrophil chemotaxis, inhibition of histamine release and cytokine-induced reduction of nitric oxide synthase, reducing inflammation. It promotes skin health by augmenting the synthesis of protein, ceramide and keratin, and encourages keratinocyte differentiation. These properties ensure that the epidermal barrier is secure, to enhance the hydration of the skin, encouraging skin health and appearance to prevent new acne lesions.^77^ On a molecular level, niacinamide was found to inhibit interleukin (IL)-8 production through the nuclear factor kappa B (NF-κB) and mitogen-activated protein kinase pathways in a dose-dependent mechanism in an *in vitro* keratinocyte/*C. acnes* model of inflammation.^78^ It appears that niacinamide is more effective in oily skin types vs. non oily skin types.^79,80^ Minor side effects of topical niacinamide include itching, burning, mild dermatitis and greasy skin.^81^

### Monotherapy

In a double-blind RCT (*n* = 60), participants received topical niacinamide 5% gel or clindamycin 2% gel for 8 weeks. At the end of the study, acne severity index was significantly decreased compared with baseline, but there was no significant difference between the two treatment and neither treatment caused adverse effects.^82^ A second study comparing niacinamide 4% gel and clindamycin 1% gel resulted in similar findings and, interestingly, the researchers noted that, given the emergence of microbial resistance, niacinamide might be a suitable alternative to clindamycin.^83^ Of note, a third study of 80 patients with moderate acne found no benefit of using a combination of clindamycin phosphate 1% gel with niacinamide 4% gel vs. clindamycin 1% alone.^84^

### Combination

In a double-blind RCT of 88 individuals, a topical adapalene/BPO (A/BPO) formulation plus a dermocosmetic regime was compared with A/BPO plus routine skincare regimen. The dermocosmetic regimen consisted of a cleanser and a cream. The dermocosmetic cleanser (Effaclar^®^ H Iso Biome cream wash; La Roche-Posay Laboratoire Dermatologique, La Roche-Posay, France) contains *Bixa orellana* seed extract, niacinamide 2%, mannose and APF (‘Aqua Posae Filiformis’). The dermocosmetic cream (Effaclar^®^ H Iso Biome cream) contains *B. orellana* seed extract, niacinamide 2%, panthenol and the pre- and postbiotic APF. There was a statistically significant difference in skin sensitivity in the dermocosmetic regime compared with the routine skincare regime, and clinical sign and symptom scores were more reduced in the dermocosmetic regimen vs. the routine skincare group, even though acne severity improved in both groups. This demonstrates the potential for niacinamide in combination with retinoids to improve treatment adherence and, hence, efficacy as the side effects of retinoids can be a treatment barrier.^85^ These findings were replicated in a second study in which a cream formulation with 8% omega-ceramides, hydrophilic sugars and niacinamide 5% reduced xerosis and skin irritation associated with oral isotretinoin and led to an improvement in adherence after 6 months of treatment.^86,87^ A retrospective assessment of three different topical combinations (*n* = 90) (group 1, BPO 5%, twice daily; group 2, BPO 5% and erythromycin 3% twice daily; group 3, niacinamide 4%, gallic acid 1% and lauric acid 1% twice daily) found no significant difference between the three groups.^88^

A new gel formulation that included combinations of retinol, encapsulated in glycospheres, and hydroxypinacolone retinoate, associated with an anti microbial peptide (BIOPEP-15) SA, GA and niacinamide has shown promising results in patients with mild acne without the side effects such as excess xerosis associated with retinoids.^11^ Further research is required to establish optimal concentrations, doses and treatment duration for topical niacinamide preparations. Direct comparison of different concentrations is required to establish robust clinical guidelines. Future research should establish appropriate treatment duration as most current research has endpoints at 8 weeks. Many of the current studies comparing niacinamide with clindamycin and erythromycin provide low-quality evidence, and methodologically robust head-to-head trials are required.^73^ In terms of future application, niacinamide is a useful adjunct to existing acne treatments. Its skin-lightening effects with melanosome transfer inhibition from melanocytes to keratinocytes could prove useful in postinflammatory hyperpigmentation.^89^

## Zinc

Zinc is a micronutrient and its role in acne vulgaris treatment has not fully been elucidated. Possible mechanisms of action were summarized by Cervantes *et al.*^90^ as regulation of protein, lipid and nucleic acid metabolism, and regulation of gene transcription for histone deacetylation. It has been suggested that zinc has a role in the regulation of DNA and RNA polymerases involved in cell replication and wound repair by acting on macrophage, neutrophils, natural killer cells and complement activity.

### In vitro

Zinc possesses anti-inflammatory activity^91^ by inhibiting IL-6 and tumour necrosis factor-α production, and inhibition of nitrous oxide, integrin and TLRs, direct inhibition of *C. acnes* and anti androgenic activity by inhibiting 5α-reductase.^90^ Given the number of patients carrying resistant *C. acnes* strains, alternative agents to antibiotics are required, and zinc oxide and silver nanoparticle concentrations of 3.9–62.5 µg mL^–1^ and 15–62.5 µg mL^–1^, respectively, significantly inhibited the growth and biofilm formation of all *C. acnes* strains.^92^ In one study, mice with a diet poor in zinc had worse disease scores, whereas those with sufficient and high zinc in their diet had better scores.^93^ Original gel formulations of zinc complexes of amino acids have been formulated with glycine and histidine, and were found to have low *in vitro* cytotoxic potential for keratinocytes and fibroblasts but had high activity against *C. acnes*. These compounds will require further *in vivo* testing to assess their clinical capabilities.^94^

### Human studies

Some patients with acne have been found to have lower serum zinc concentrations, but correlations of disease severity and type of acne are conflicting.^95^ A systematic review and meta-analysis of 2445 patients found that patients with acne vulgaris had significantly lower mean (SD) levels of zinc compared with controls [96.308 (4.053) μg dL^–1^ in acne vs. 102.442 (3.744) μg dL^–1^ in control participants; *P* = 0.041], and zinc treatment subsequently improved the mean inflammatory papule count vs. the nontreatment group as monotherapy or in combination.^96^ In a separate study, oral zinc combined with low-dose isotretinoin was compared with isotretinoin alone in 60 patients with acne vulgaris. No significant difference was found in the efficacy of treatment between groups in terms of lesion count and IGA scores. However, there were fewer side effects reported in the combined treatment group suggesting that combined treatment could result in better treatment adherence.^97^ Significant reductions in noninflammatory lesion counts were found in patients treated with a formulation of niacinamide cream, antibacterial adhesive and zinc-pyrrolidone carboxylic acid and adapalene compared with adapalene alone after twice daily treatment for 6 weeks (*n* = 140), with no significant differences found in the side effect profile.^98^ Combinations of topical erythromycin 2% with zinc acetate 1.2% vs. erythromycin 2% monotherapy was compared in patients with mild-to-moderate acne over 3 weeks (*n* = 102). There was a reduction in lesion count and severity in the combination treatment group, although this was not statistically significant.^99^ A recent single-centre open-label nonrandomized study (*n* = 51) investigated the use of a cleansing gel containing SA 2%, zinc gluconate 0.2% and lipohydroxy acid 0.05% in moderate truncal acne. Total lesion count was significantly reduced after 84 days, as was noninflammatory lesion count, with good product tolerability.^100^

## Tea tree oil

Tea tree oil is derived from *Melaleuca arternifolia* leaves and branches. Its anti-inflammatory and immune properties mean it is useful in a variety of dermatological conditions. Terpinen-4-ol is one of the major antimicrobial and ­anti-inflammatory components of tea tree oil.^101^

### In vitro

Tea tree oil has found to have broad antimicrobial and antibiotic effects *in vitro* and has been shown to be effective against *C. acnes*.^102^ The synergistic effects of an azelaic acid and a tea tree oil microemulsion hydrogel were explored *in vitro* to assess its efficacy against the bacterial strains *Staphylococcus aureus*, *C. acnes* and *Staphylococcus ­epidermidis*, and was found to increase the zone of inhibition by twofold and decrease the minimum inhibitory concentration by eightfold against *C. acnes* vs. azelaic acid alone. It also reduced papule density more than azelaic acid alone and had better permeation, retention, skin-compliant attributes and texture.^103^

### Human studies

In a single-blind RCT (*n* = 124), tea tree oil 5% gel was compared with BPO 5% lotion in patients with ­mild-to-moderate acne. Both therapies significantly reduced the number of inflammatory and noninflammatory lesions. Although the tea tree oil took longer to exhibit these effects, it had a better side effect profile and, at the end of 3 months, there were no differences seen between the two therapies.^104^ The side effects of tea tree oil include skin irritation, contact dermatitis, erythema multiforme-like reactions and idiopathic prepubertal gynaecomastia, with oral poisoning reported at higher doses.^101^ In one RCT, tea tree oil combined with adapalene gel once daily (ADA Marketed Gel) was compared with adapalene 0.1% gel once daily (*n* = 100). There was a significantly greater reduction in total, inflammatory and noninflammatory acne lesion count in the combined group compared with the monotherapy group (*P* < 0.001) after 12 weeks. Dry skin was the most common side effect and this was higher in the treatment group.^105^ Tea tree oil combined with propolis and aloe vera was found to be more effective than topical erythromycin in reducing erythema scars, acne severity index and total lesion count in a double-blind trial of 60 patients with mild-to-moderate acne.^106^ In a small study of 18 participants, tea tree oil face wash (7 mg g^–1^) and gel (200 mg g^–1^) were found to significantly reduce total lesion counts (*P* < 0.0001) and reduced mean IGA score after 12 weeks (*P* = 0.0094). Reported side effects included peeling, dryness and scaling, which resolved independently.^107^ Overall, it is difficult to robustly support the use of tea tree oil in acne vulgaris. Most studies are small or *in vitro* and use different formulations with other active ingredients with different end outcomes, making it difficult to find a true relationship between tea tree oil and its anti-inflammatory effect on acne.^108^

## Green tea

Green tea is an important source of plant polyphenols and is composed of catechins, flavonols and flavonoids. Green tea has the highest components of EGCG, which is thought to act on the acne signalling cascade involving insulin growth factor-1 (IGF-1; thought to play a role in Western-diet induced acne).^109^

### In vitro

Catechins in green tea are thought to have ­anti-inflammatory and antimicrobial properties, while polphenon-60 (a derivative of green tea extract) inhibited IL-8 secretion and TLR-2 expression stimulated by *C. acnes* by downregulating extracellular signal-regulated kinases 1/2 and AP-1 pathways in *ex vivo* studies of human monocytic cells,^110^ and has been found to reduce comedones and pustules in ­mild-to-moderate acne.^111^  *In vitro* studies have also shown that green tea extract can inhibit lipid synthesis in IGF-1-treated sebocytes and suppress the release of inflammatory cytokines. Androgens are thought to play a key role in the acne pathway and green tea extract was found to reduce 5α-reductase activity, and therefore testosterone, decreasing sebum synthesis in hamster sebocytes.^112^ Green tea extract has antimicrobial activity against *C. acnes*, *Propionibacterium granulosum*, *S. aureus* and *S. epidermidis*,^113^ and exhibits activity against free radicals, diminishing oxidative stress and the inflammatory response by suppressing the NF-κB pathway.^113^

### Human studies

Five RCTs were included in a systematic review and meta-analysis (*n* = 247). Green tea 5% extract significantly reduced the number of inflammatory lesions (−9.38, 95% CI −14.13 to −4.63), while subgroup analysis showed that topical green tea extract significantly reduced inflammatory lesion counts (−11.39, 95% CI −15.91 to −6.86). However, oral green tea had a minimal impact (−1.40, 95% CI −2.50 to −0.30) on inflammatory lesion count. Non-inflammatory lesions were not reduced by green tea extract, although when this was stratified by route of delivery, topical green tea extract significantly reduced noninflammatory lesions (−32.44, 95% CI −39.27 to −25.62), but oral administration did not (0.20, 95% CI 0.00–0.40).^114^ Topical green tea extract was shown to be as efficacious as clindamycin 1% and superior in treating erythema and pigmentation. Mild gastrointestinal effects were reported relating to oral green tea extract and temporary irritation, and itching was associated with topical green tea extract application.^115^ A second systematic review of eight studies explored the evidence for green tea and polyphenols in sebum production in acne vulgaris.^109^ They found some evidence for topical tea polyphenols in reducing sebum secretion in acne vulgaris. However, some studies included were not explicit in the type of tea used or the amount of tea polyphenol in each tea sample. Many of the included studies had poor research designs lacking control groups, and therefore a quantitative pooled effect size was not generated. A single-blind placebo study of 60 women with mild-to-moderate acne investigated the effect of sonophoresis with an ultrasound gel combined with green tea, bamboo extract and lactic acid vs. ultrasound gel alone. The treatment had a significant effect on reducing facial greasing around the nose compared with placebo, but there was no statistically significant difference effect in levels of greasing between the eyebrows and bottom lip. There were no significant adverse effects reported. In future studies, it would be useful to include pH and skin moisture as additional skin parameter measures with a more diverse group of patients, with an appropriate treatment duration investigated. In addition, it would be useful to compare the effect of the treatment with and without sonophoresis.^116^

In one RCT, 47 patients with acne vulgaris were randomly assigned to receive tea lotion 2% or zinc sulfate 5% solution for 2 months, with a significant reduction in inflammatory lesion count (pustules and papules) noted in the tea group, with no significant difference noted in the zinc sulfate group.^117^ In human studies, it appears that oral green tea extract exerts its activity on inflammatory rather than noninflammatory lesions. A herbal extract-loaded hydrogel containing green tea, *Zingiber officinale* Rosc., *Phyllanthus emblica* and SA demonstrated moderate-to-high healing properties as a bioactive dressing agent in 24 patients with acne.^118^ In a small split-body study, green tea 5% was compared with placebo, and green tea 2.5% and lotus 2.5% extract for 60 days (*n* = 22). The green tea (27% reduction, *P* = 0.006) and green tea plis lotus group (25% reduction *P* = 0.002) both had significant reductions in their sebum production (measured with a Sebumeter^®^; Courage + Khazaka electronic, Köln, Germany), with the combination group experiencing greater sebum secretion reduction than the group using green tea alone, although it must be noted that these patients were healthy and did not have acne.^119^ In a separate study, green tea 3% extract used for 8 weeks led to a sebum reduction of 60% in 10 healthy control individuals (*P* < 0.095).^120^ These positive findings were replicated in two separate studies comparing tea 2% lotion with placebo.^121,122^ Overall, all studies investigating the use of green tea extract have small sample sizes without comparative outcome measures, and have differing treatment durations and concentrations, with some studies containing qualitative assessment measures. Whether there was a recurrence in acne lesions was usually not investigated.^114^

## Conclusion

Cosmeceuticals may be useful adjuncts in the treatment of acne vulgaris, with their anti-inflammatory and antimicrobial properties. Research suggests that some cosmeceuticals may have similar efficacies to established treatments such as clindamycin and topical retinoids.

Given that treatments such as isotretinoin may have undesirable side effects or may be contraindicated, cosmeceuticals may afford a more accessible alternative, improving patient compliance and treatment outcomes. Cosmeceuticals can be adjunctive agents used to ameliorate acne complications such as scarring and postinflammatory hyperpigmentation.

BPO remains a key over-the-counter agent with robust evidence for inflammatory acne, while cosmeceuticals such as retinol, niacinamide and azelaic acid may be most beneficial for patients with mild-to-moderate acne, sensitive skin or those unable to tolerate prescription treatments. However, few robust studies address the role of cosmeceuticals in diverse skin types, severe acne, long-term use or head-to-head comparisons with prescription agents. While this review prioritized high-level evidence, it is important to acknowledge that many included studies had methodological limitations. Several RCTs lacked blinding, or had small sample sizes or short follow-up periods, limiting their external validity. Combination formulations were frequently studied, making it difficult to isolate the effect of individual cosmeceutical ingredients. Additionally, some trials were industry-sponsored, possibly introducing bias. Additionally, the heterogeneity of formulations, dosing and outcome measures also complicates direct comparison across studies and limits the ability to draw robust conclusions.^123^ Additionally, vehicle formulation might impact the tolerability and effectiveness of cosmeceuticals. For example, lipid-based nanocarrier vehicles have been shown to improve retinol delivery,^124^ while a microsphere formulation of BPO reduced irritation compared with a gel formulation.^125^ Future research should stratify outcomes by formulation to identify optimal delivery systems. Robust studies are required to demonstrate the safety, effectiveness and quality control of cosmeceuticals, which are available over the counter with less stringent guidelines for their use and manufacturing standards. Standardized outcome measures such as IGA and DLQI, and adequate follow-up durations are needed to allow rigorous comparison with current gold standard treatments. There are large evidence gaps for severe acne, including in paediatric and older patients, and those with Fitzpatrick IV–VI skin types. Head-to-head trials with prescription agents and the inclusion of underrepresented patient subgroups should be encouraged. Overall, there is most robust evidence to support the use of topical retinoids,^126^ BPO and azelaic acid in the treatment of mild-to-moderate acne vulgaris. There is mostly low-quality evidence to support the use of the other cosmeceuticals discussed and data comparing these treatments to established prescription treatments are scarce. We acknowledge that our review is not exhaustive and there are multiple other cosmeceutical agents that have evidence of utility, including (but not limited to) silymarin,^127^ decanediol,^128^  L-carnitine^129^ and sulfur.^130^

## Data availability

No new data were generated or analysed in support of this research.
